# Unveiling the hidden culprit: Parathyroid adenoma induced recurrent renal calculus and pancreatitis—A case report

**DOI:** 10.1002/ccr3.9248

**Published:** 2024-08-05

**Authors:** Aakash Kumar Pandit, Prajjwal Pokharel, Kabin Sapkota, Sanket Dhakal, Ram Narayan Kurmi, Mukesh Kumar Ranjan

**Affiliations:** ^1^ Department of Gastroenterology and Hepatology Chitwan Medical College Bharatpur Nepal

**Keywords:** hypercalcemia, pancreatitis, parathyroid adenoma, primary hyperparathyroidism, renal stones

## Abstract

This case report describes a 52‐year‐old patient presenting with recurrent episodes of pancreatitis and renal stones. Further investigation revealed hypocalcemia and elevated parathyroid hormone (PTH) levels, leading to diagnosis of a parathyroid adenoma. This case highlights the importance of considering primary hyperparathyroidism in patients with recurrent pancreatitis and renal stones, as early diagnosis and surgical intervention can prevent recurrence and reduce morbidity.

**Abstract:**

Primary Hyperparathyroidism secondary to Parathyroid adenoma, rarely presents as acute pancreatitis. A 38‐year‐young male with a history of recurrent renal stones referred from a local center, presented to the emergency services, with a diagnosis of acute pancreatitis and bilateral renal stones. Laboratory evaluation showed an elevated calcium level, elevated PTH levels, low vitamin D, and low phosphorus levels. CT scan done outside was suggestive of acute pancreatitis along with bilateral renal calculi. USG neck and MIBI scan done as a part of hypercalcemia evaluation showed presence of a right parathyroid adenoma. Parathyroid adenoma was later removed, and calcium and parathyroid levels were normal on subsequent follow ups.

## 1 INTRODUCTION

Acute pancreatitis includes inflammation and injury to the pancreas, which may lead to sepsis and multi organ dysfunction.^1^ Pancreatitis is considered as a major public health concern worldwide and the incidence has increased in the last 30 years, with the highest increment seen in South Asia.^1^ Gall stones and alcohol contribute as the major etiological factor of acute pancreatitis, together contributing approximately 2/3rd of the total cases.^2^ Hypercalcemia is an uncommon cause of pancreatitis that causes calcium deposition in the pancreatic duct and calcium activation of trypsinogen.^3^ Hypercalcemia classically presents as bone pain, constipation, renal stones, fatigue and neurological disturbances such as depression and confusion. Primary hyperparathyroidism is a common cause of hypercalcemia, and usually results due to a solitary parathyroid adenoma, accounting to around 80%–85% of the total cases of primary hyperparathyroidism.^4^ However, parathyroid adenoma presenting as acute pancreatitis is a rare condition, contributing to around 1.5%–7% of the total etiology of acute pancreatitis.^5^, ^6^ Only few cases of pancreatitis secondary to hypercalcemia are reported,^7^ which can lead to bias towards the diagnosis. Here we present a case of acute pancreatitis and bilateral renal stones due to hypercalcemia secondary to a parathyroid adenoma.

## 2 CASE REPORT

### 2.1 Case history

A 38‐year young male with a history of occasional alcohol consumption and recurrent renal stone formation, was referred to the emergencies services of Chitwan Medical College (CMC) with a provisional diagnosis of acute pancreatitis. This was his second episode of pancreatitis in the past 4 months. He had chief complaints of pain in the epigastrium and left upper quadrant of the abdomen, nausea, and vomiting. At presentation, patient was found to have sinus tachycardia and systemic hypertension [Systolic blood pressure 135–150 mmHg; diastolic blood pressure: 90–100 mmHg]. His pulse was 106 bpm, respiratory rate was 26/min, SpO_2_ was 96% in room air, and he was afebrile during presentation.

### 2.2 Methodology

**FIGURE 1:**
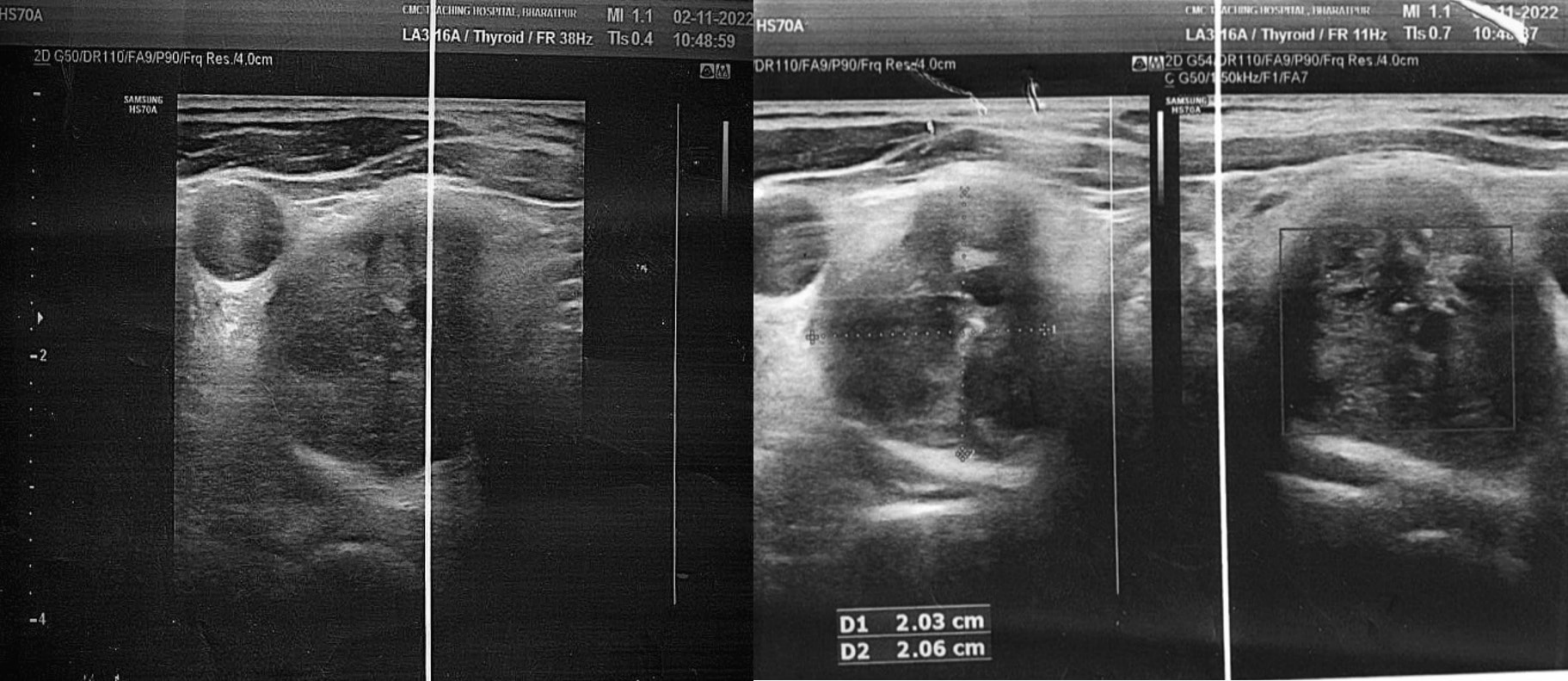
USG of the parathyroid gland showing a well‐defined heterogeneous lesion of 20 × 23 mm with nodular outline showing few areas of calcification noted separately from the right lobe of thyroid lying posteroinferior to it.

**FIGURE 2:**
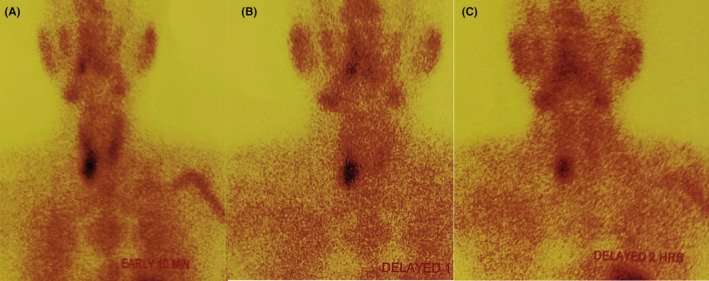
Technitium‐99 m Sestamibi scan showing retention areas near the inferior pole of the right thyroid gland (A) Early 10 min (B) Delayed 1 h (C) Delayed 2 h.

**FIGURE 3:**
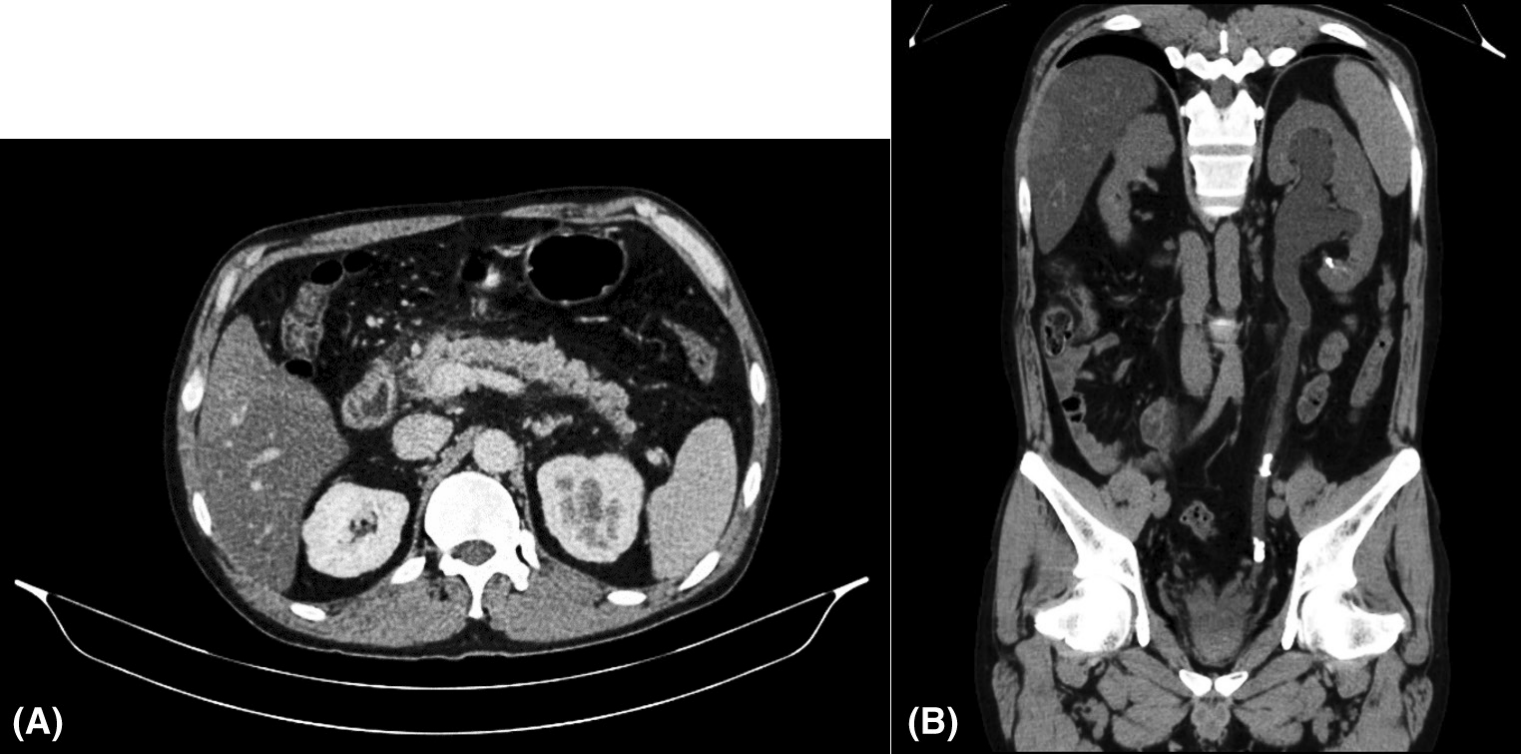
CT scan (Abdomen + Pelvis) suggestive of focal interstitial pancreatitis (A) and left sided ureteric stone (B).

Available laboratory reports from previous hospital showed elevated amylase levels, 286 U/L (range, 20–80 U/L), elevated lipase levels, 1176.0 U/L (range, 13–60 U/L). CT scan which was done outside and reviewed at our center in the department of radiodiagnosis was suggestive of acute pancreatitis (CTSI = 2/10) with bilateral renal stones with moderate left hydronephrosis (Figure 3). No secondary cause for hypertension was identified on evaluation. The patient was admitted to the ICU for further management. In the ICU, he was kept nil per oral for 48 hours, and ringer lactate and dextrose saline was given IV at the rate of 120 mL/h. The patient improved symptomatically with these conservative managements, and gradually solid foods were started Similarly, IV Tramadol 50 mg and IV Ondansetron 4 mg were used as analgesics and antiemetic. He was started on Tab Amlodipine 5 mg following transfer to the Gastroenterology ward. Other work‐up showed an elevated triglyceride level, 187.73 mg/dL (65–170 mg/dL), elevated VLDL, 37.55 mg/dL (range, 13–34 mg/dL), elevated calcium levels, 12.9 mg/dL (range, 9–11 mg/dL), elevated iPTH levels, 105.9 pg/mL (range, 18.5–88 pg/mL), low vitamin D, 11.22 ng/mL (range, deficiency <20 ng/mL) and low phosphorus levels, 2.2 mg/dL (2.5–5 mg/dL). As a part of hypercalcemia evaluation, USG neck revealed features of parathyroid adenoma (Figure 1). Technetium‐99 m Sestamibi scan was performed which was suggestive of a right parathyroid adenoma (Figure 2). Baseline evaluation showed derranged liver pannel tests. The test results are mentioned in Tables 1 and 2. For a possible cause of biliary pancreatitis, USG evlauation and CT discussion did not revealed any biliary pathology. The liver pannel tests remained elevated at day five of the patient admission, suggestive possibly of acute hepatitis.

**TABLE 1:** Blood Investigation Reports.

Test	Result	Reference
Hemoglobin (Hb)	15.1	11.0–16.0 g/dL
WBC	10,500	4000‐11,000/mm^3^
Platelets	255,000	150,000‐400,000/mm^3^
Urea	34.0	10–50 mg/dL
Creatinine	0.68	0.4–1.4 mg/dL
Na^+^/K^+^	139.0/4.3	(135–155)/ (3.5–5.5) mmol/L
LFT		
Bilirubin (total)	**2.65**	<2.0 mg/dL
Bilirubin (direct)	**1.16**	<0.4 mg/dL
SGOT/SGPT	**123.0/94.0**	(10–50)/ (10–50) U/L
Serum ALP	**204.0**	50.0–136.0 U/L
Total Protein	6.5	6.0–8.5 g/dL
Serum Albumin	4.5	3.7–5.3 g/dL
PT/INR	1.06	
Serum amylase	**286.0**	20–80 U/L
Lipase	**1176.0**	13–60 U/L
Calcium	**12.9**	9–11 mg/dL
Triglycerides	187.73	65–170 mg/dL
LDL/VLDL	49.97	<150/ (13–34) mg/dL
Phosphorus	**2.2**	Adult: 2.5–5.0 mg/dL
Vitamin D (25‐OH), Serum	**11.22**	Deficiency: <20 ng/mL
Intact Parathyroid Hormone, Serum	**105.9**	18.5–88.0 pg/mL

**TABLE 2:** Urine investigation reports.

Test	Reports	Reference
Routine test		
Blood (Free Hb, myoglobin, RBCs)	++	
Ketone	+	
Bilirubin/glucose/ascorbic acid	Nil	
Microscopic examination		
Pus cells	6–8	/HPF
Epithelial cells	2–4	/HPF
RBCs	**Plenty**	/HPF
Urinary calcium	12.3	mg/dL
Urinary creatinine	37.77	mg/dL
Urine calcium/creatinine ratio	**0.32**	<0.14

## 3 RESULTS

Urosurgery team was consulted, and the patient was planned for surgical removal of the renal calculus. Department of ENT was also consulted, and the patient was also planned for the surgery for parathyroid adenoma. Parathyroid adenoma removal was done, and the surgery had no complications. Upon subsequent follow ups, the patient had normal calcium and parathyroid levels. He was doing well, and he had no complaints of any features suggestive of the recurrence of the previous diseases.

## 4 DISCUSSION

In this case report, we present a case of acute pancreatitis and recurrent renal stones due to parathyroid adenoma resulting in hypercalcemia. Hypercalcemia accounts to around 1.5%–8% of the total cases of pancreatitis and most of which is associated with hyperparathyroidism.^7^ Hypercalcemia in a setting of recurrent renal stones requires thorough investigation and is strongly suggestive of primary hyperparathyroidism.^8^ Cope et al was the pioneer to bring out the concept of pancreatitis due to hyperparathyroidism with his article in the Annals of Surgery in 1957.^9^ Mayo clinic in between 1950 and 1975, operated 1153 patients with hyperparathyroidism and reported only 17 individuals (1.5%) to have a coexisting pancreatitis.^10^ Prinz et al in a study found only 0.4% of the cases of acute pancreatitis to be caused by hyperparathyroidism.^11^ Similarly, Carnaille B et al in 1998 retrospectively evaluated 1224 cases of hyperparathyroidism and found that acute pancreatitis was present in 3.2% cases.^12^ The questions thus arise about the relation of hyperparathyroidism and acute pancreatitis and the controversies that follow through.^3^ Sitges‐Sara in 1998, has however suggested the causation of pancreatitis due to hypercalcemia, but due to nonparathyroid causes and criticized the reports published by Mayo Clinic to not be true.^13^ Amid the controversy, Abdhullah M et al in 2003 reported a case of recurrent pancreatitis with elevated serum calcium and elevated parathyroid hormones leading to the surgical exploration of neck to find a solitary parathyroid adenoma.^13^ Despite the debate, it is still recommended to have the parathyroid levels checked in the setting of recurrent pancreatitis.^3^ However, calcium stones in the form of calcium oxalate and calcium phosphate contribute the majority (85%) to the cause of renal stones and a history of such may be indicative of hypercalcemic state and may require further evaluation.^8^, ^14^ Thus, a clinical suspicion arose due to the history (2nd episode of pancreatitis within 4 month) and investigations of the patients which eventually led to the diagnosis of parathyroid adenoma leading to a hypercalcemic state presenting as a combination of acute pancreatitis and bilateral renal calculus (Figure 3).

Acute pancreatitis in a setting of hyperparathyroidism occurs either due to hypercalcemic state leading to de novo activation of trypsinogen to trypsin, leading to auto digestion, or due to the ductal obstruction secondary to formation of ductal calculi, or due to the genetic risk factors that may predispose patients with hyperparathyroidism to acute pancreatitis.^5^

Acute pancreatitis is self‐limiting in most cases. Management consists of IV fluids, analgesia, and enteral feeding in those able to tolerate. Treatment focuses on the management of the underlying condition. Parathyroid adenoma causing hypercalcemia should thus be operated for the definitive management of such cases. Without the removal of the PTH secreting adenoma, the symptoms will reappear in the future, and thus any other forms of management will only be symptomatic relief for the time being. USG or Technetium‐99 m Sestamibi scan may be useful in the location of pathology within the parathyroid gland.^15^

It is seen that parathyroidectomy seems to prevent the recurrent attacks of pancreatitis and nearly 100% improvement in pancreatitis has been seen following the treatment of hyperparathyroidism but the cases of renal calculus following parathyroidectomy have seen to increase within the immediate years which declines later signifying an overall benefit of the treatment.^16^, ^17^ Our patient also had systemic hypertension for which no obvious cause was found in the evaluation. Hypertension is reported to be present in 40–60% of patients with primary hyperparathyroidism.

Hypercalcemia secondary to parathyroid adenoma is a relatively uncommon cause of acute pancreatitis. Recurrent renal stones in presence of hypercalcemia should lead to evaluation for parathyroid adenoma in patients with acute pancreatitis.

## 5 CONCLUSION

This case report highlights the diagnostic challenges and management considerations in a patient presenting with primary hyperparathyroidism secondary to a parathyroid adenoma. Through a comprehensive clinical evaluation, including biochemical tests and imaging modalities such as ultrasound and sestamibi scintigraphy, the diagnosis of parathyroid adenoma was established. Surgical resection of the adenoma led to the normalization of serum calcium and parathyroid hormone levels, thereby reducing the risk of long term complications such as osteoporosis.

This case contributes to the existing literature by providing insights into the clinical presentation, diagnostic approach, and therapeutic interventions for parathyroid adenoma. Further studies are warranted to explore emerging diagnostic modalities and refine treatment strategies for this common endocrine disorder.

## AUTHOR CONTRIBUTIONS

**Aakash Kumar Pandit:** Data curation; formal analysis; investigation; writing – original draft; writing – review and editing. **Prajjwal Pokharel:** Conceptualization; data curation; formal analysis; resources; writing – original draft; writing – review and editing. **Kabin Sapkota:** Investigation; methodology; software; writing – review and editing. **Sanket Dhakal:** Investigation; methodology; resources; writing – original draft; writing – review and editing. **Ram Narayan Kurmi:** Conceptualization; supervision; validation. **Mukesh Kuamr Ranjan:** Conceptualization; project administration; supervision; validation; writing – review and editing.

## FUNDING INFORMATION

The authors declare that they have no known competing financial interests or personal relationships that could have appeared to influence the work reported in this paper.

## CONFLICT OF INTEREST STATEMENT

None.

## ETHICS STATEMENT

The authors declare that the procedures were followed according to the regulations established by Clinical Research and Ethics Committee and to the Helsinki Declaration of the World Medical Association updated in 2013.

## CONSENT

Written informed consent was obtained from the patient to publish this report in accordance with the journal's patient consent policy.

## Data Availability

The datasets analyzed during the current study are available from the principal and the corresponding authors upon reasonable request. Additionally, comprehensive literature sources used for the literature review are cited appropriately, within the manuscript.
